# An automated method for developing search strategies for systematic review using Natural Language Processing (NLP)

**DOI:** 10.1016/j.mex.2022.101935

**Published:** 2022-11-23

**Authors:** Antwi Effah Kwabena, Owusu-Banahene Wiafe, Boakye-Danquah John, Asare Bernard, Frimpong A.F. Boateng

**Affiliations:** a Canadian Forest Service, Great Lakes Forestry Centre, 1219 Queen Street East, Sault Ste. Marie, Ontario, P6A 2E5; b University of Ghana, Department of Computer Engineering, P.O. BOX LG 77, Legon, Accra, Ghana

**Keywords:** Search Strategy, Search Terms, Data Deduplication, Software Implementation, Evidence Synthesis, Systematic Literature Review

## Abstract

The design and implementation of systematic reviews and meta-analyses are often hampered by high financial costs, significant time commitment, and biases due to researchers' familiarity with studies. We proposed and implemented a fast and standardized method for search term selection using Natural Language Processing (NLP) and co-occurrence networks to identify relevant search terms to reduce biases in conducting systematic reviews and meta-analyses.

• The method was implemented using Python packaged dubbed *Ananse, which is* benchmarked on the search terms strategy for naïve search proposed by Grames et al. (2019) written in “R”. *Ananse* was applied to a case example towards finding search terms to implement a systematic literature review on cumulative effect studies on forest ecosystems.
• The software automatically corrected and classified 100% of the duplicate articles identified by manual deduplication. *Ananse* was applied to the cumulative effects assessment case study, but it can serve as a general-purpose, open-source software system that can support extensive systematic reviews within a relatively short period with reduced biases.
• Besides generating keywords, *Ananse* can act as middleware or a data converter for integrating multiple datasets into a database.

## Graphical abstract

**Figure:**
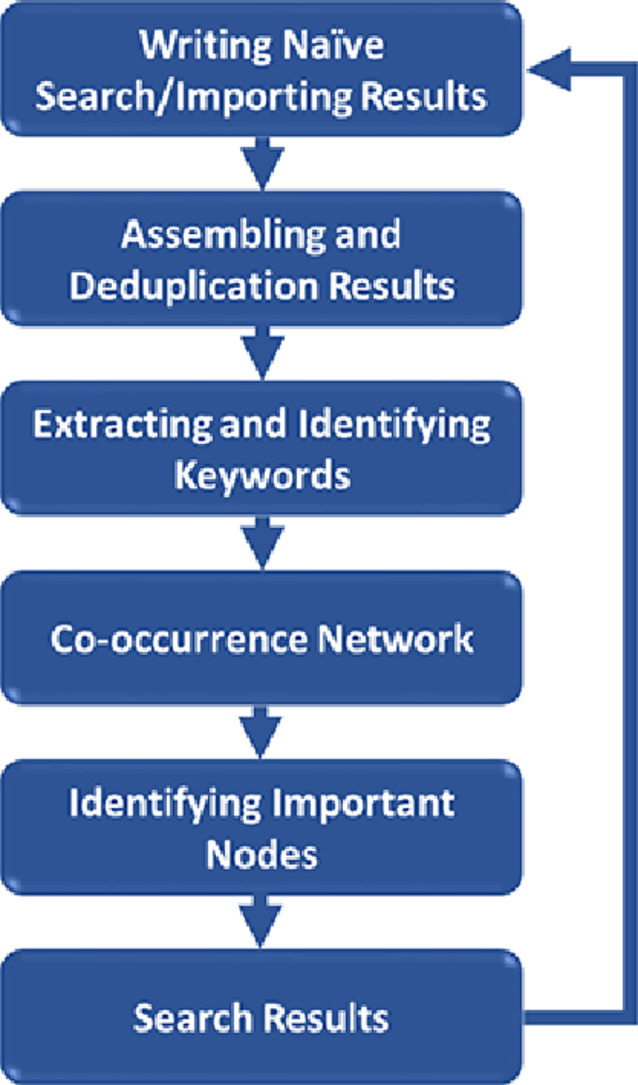

Specifications table

**Table:** 

Subject Area	Environmental Science
More specific subject area	*Evidence synthesis in environmental and biological sciences*
Method name	*Text mining and keyword co‐occurrence networks to identify the most important terms for a review*
Name and reference of original method	Grames, E. M., Stillman, A. N., Tingley, M. W., & Elphick, C. S. (2019). An automated approach to identifying search terms for systematic reviews using keyword co‐occurrence networks. *Methods in Ecology and Evolution, 10(10), 1645-1654.*
Resource availability	*Documentation:*https://baasare.github.io/ananse/_build/html/index.html *Software:*GitHub - baasare/ananse Method description: ananse · PyPI

## Background

Historically, summaries of scientific evidence have helped discover patterns of phenomena, develop theories or concepts, and inform practice. Although common with editors and readers alike, this approach is less rigorous since evidence summarized this way is less likely to answer specific clinical questions and more likely to contain literature selected by the authors and recommendations prejudiced strongly by opinion. With exponential growth in scientific literature, the search for a structured and effective evidence synthesis has become a critical scientific endeavor. Evidence synthesis involves combining information from multiple studies or research that have investigated the same or similar issue to come to a conclusive understanding of a specific topic [1]. It often involves summarizing trends, identifying emerging questions, and clarifying disagreements and conflicting results [2,3].

Since 1753 when James Lind published the first evidence synthesis to provide a concise and unbiased summary of evidence on scurvy, improvement in the state of evidence synthesis has grown [4,5]. In the past two decades, advances in computer-aided technology have enabled the growth and development of various forms of evidence synthesis. The two central techniques known to have originated from the medical sciences and are commonly used today to synthesize evidence are systematic reviews (SRs) – which search available literature for evidence that addresses the research question, - and meta-analyses – which quantitatively assess statistical evidence found through systematic reviews [5]. Evolutionary and behavioral ecologists started adopting meta-analyses in the mid-1990s and became fully embraced since 2010 [6]. Meta-analysis has since become the gold standard for combining information from multiple studies across disciplines. However, a good meta-analysis is dependent on a good sampling of the core universe of studies, thus requiring a careful and comprehensive SR. A SR involves the review of an articulated research question using systematic and testable methods to help to identify, select and evaluate all pertinent research [7], and collect and analyze data from the studies that are included in the review [8]. An excellent SR assembles and presents an impartial and objective summary of findings, assesses all results for inclusion/exclusion and quality, and minimizes bias at all stages of the process [7].

However, the process of evidence synthesis is very tedious and often involves experienced methodologists and disciplinary experts combing through all relevant studies, both published and unpublished, through a guided methodological process. As such, it tends to be costly and tedious as it can take months, or even years, to complete, making it practically challenging [9]. According to some estimates, conducting a SR can take up to 2 years to complete. [10] also suggest that the time needed to complete a SR with meta-analyses ranges from 216 to 2,518 hours. According to [11], conducting an effective systematic search requires an information specialist's expertise and time, who need an average aggregated time of 26.9 hours when developing a search strategy. Thus, the design and implementation of evidence-based synthesis are hampered by high financial costs [3] and significant time commitment [2].

To overcome time and resource constraints required to synthesize evidence, scholars have adopted automation of the laborious tasks in SR [12]. Advances in computer-aided technology have helped automate aspects of the evidence synthesis process to improve efficiency and cut costs and time while still maintaining the standards of conventional search methods [13]. Automation occurs in different forms; from the most basic of tasks to complicated ones [13], such as removing duplicate articles, prioritizing articles for screening, and extracting data from tables and figures [14,15]. Research on different approaches for automating systematic reviews via technologies such as machine learning, text mining, and natural language processing exists [12]. Text mining is the process of discovering knowledge and structure from unstructured data [16], while Natural Language Processing (NLP) supports human analysts to carry out various linguistic analytical tasks on textual documents [17], such as identifying potential keywords in systematic literature reviews [18], [19], [20]. Using NLP to extract information from text automatically leads to decreased labor of manual extraction from a large volume of text material and saves time [21].

However, automation in SR has focused chiefly on extracting data or results after a literature search, while methods or strategies to find or assemble all relevant evidence, including developing a search strategy, have received little attention [22]. According to [22], search strategies for SR should be able to return all the studies relevant to the review (‘recall’) without retrieving irrelevant studies (‘precision’). Unfortunately, not all fields of study have a structured or standardized ontology for search strategy development. The field of public health has institutionalized support and standardized ontology (i.e., Medical Subject Headers, or MeSH) for search strategy development [23]. However, ecology or environmental sciences, generally, does not have standardized ontologies. Thus, researchers tend to use broad, non‐specific keywords in their search (Pullin & Stewart, 2006), leading to low precision of search results (0.473%; [2]). With low precision, more time and cost are spent on screening articles. Thus, enhanced standardization in search strategy development is critical to improving the specificity, objectivity, and reproducibility of SRs [24]. Two primary approaches for automating search strategy development are citation networks and text mining [22]; both use a set of predetermined articles that researchers deem relevant to the review. Thus, both approaches require researchers to select a starting set of articles with which they are already familiar. This predisposes citation networks and text mining towards familiar articles. Although this approach has high precision, it has a low recall, and the risk of selection, citation, and publication bias is increased as the initial set of articles influences what is eventually retrieved [25,22,26].

In this research, we mediate the problems associated with search strategy development in systematic literature reviews by developing a method that uses NLP and keyword co-occurrence networks to identify potential keywords to support SR. We adapted the search terms strategy for naïve search proposed by [22] written in R. To facilitate reproducibility and transparency; we created the python package dubbed ‘Ananse’ (a Ghanaian vernacular translated as a spider) to aid the implementation of the method in a user-friendly format. The software and documentation are publicly available via Github [27] and PyPI [28], [29], [30], respectively. We tested our approach by applying it to selecting keywords for a systematic literature review of cumulative effect assessment of disturbance on forest ecosystems (see [30]).

The remainder of the study is structured as follows. Materials and methods are presented in section 2, where the process flow of Ananse in finding search terms are described. Using Ananse to perform a search tailored to a SR of cumulative effect studies is described in section 3. In section 4, we discuss the outcomes of using *Ananse* to perform cumulative effect search terms [30] and compare our results with other related works. Finally, in section 5, we draw conclusions based on our findings and forecast future work.

## Methods details

We developed a Python package to partially automate search term selection and write search strategies for SRs. We refer to this Python package as *Ananse* (a Ghanaian vernacular translated as a spider). We adapted the *search strategy for black-backed woodpecker occupancy of post-fire forest systems* ( [22] and [31]) written in R. Our search term selection strategy focuses on cumulative effect and seeks to create an open-source search software in Python.

### Software design

Software design describes the structure of the software to be implemented, the data models used by the system, the interfaces, and, sometimes, the algorithms used [32]. Requirements usually precede the design. We present the following design considerations during the creation of Ananse: functional requirements, use case diagram, and data flow diagram. We do not intend to offer a technical software engineering perspective but to guide the user to appreciate the design concepts which gave birth to Ananse.

#### Functional requirements

The functional requirements for a software system describe what the system should do [33,34]. We considered the SR process from the NLP perspective and specified the requirements for Ananse. Ananse is able to:

1. Import results of a naïve search from a literature database such as JSTOR, Web of Science, and Scopus just to mention a few.
2. Deduplicate combined search results.
3. Extract terms using Rapid Automatic Keyword Extraction (RAKE) algorithm
4. Create document term matrix.
5. Convert document term matrix into data frames.
6. Create document network from data frames.
7. Generate node strength and final cut-off.
8. Generate keywords.

These eight requirements were used to formulate a use case diagram.

#### Use case diagram

Use cases are documented using a high-level use case diagram. The set of use cases represents all of the possible interactions described in the system requirements. Actors in the process, who may be human or other systems, are represented as stick figures. Each class of interaction is represented as a named ellipse. Lines link the actors with the interaction; arrowheads show how the interaction is initiated.

**Fig. 1:**
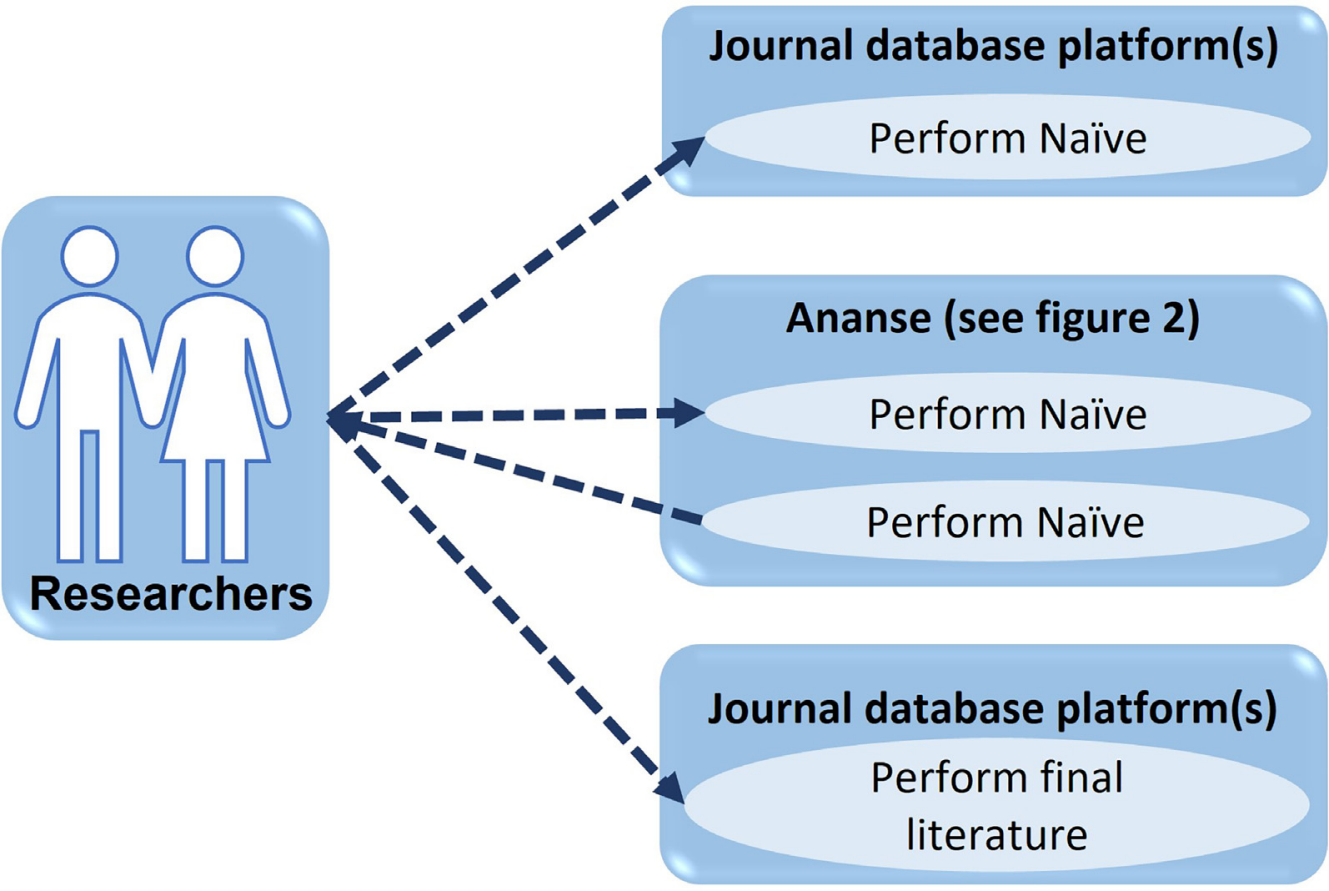
Use case diagram for *Ananse.*

Figure 1 is the use case diagram for *Ananse. A* researcher performs naïve a search from a journal database platform such as Web of Science, Scopus, or JSTOR.

#### Flow diagram

**Fig. 2:**
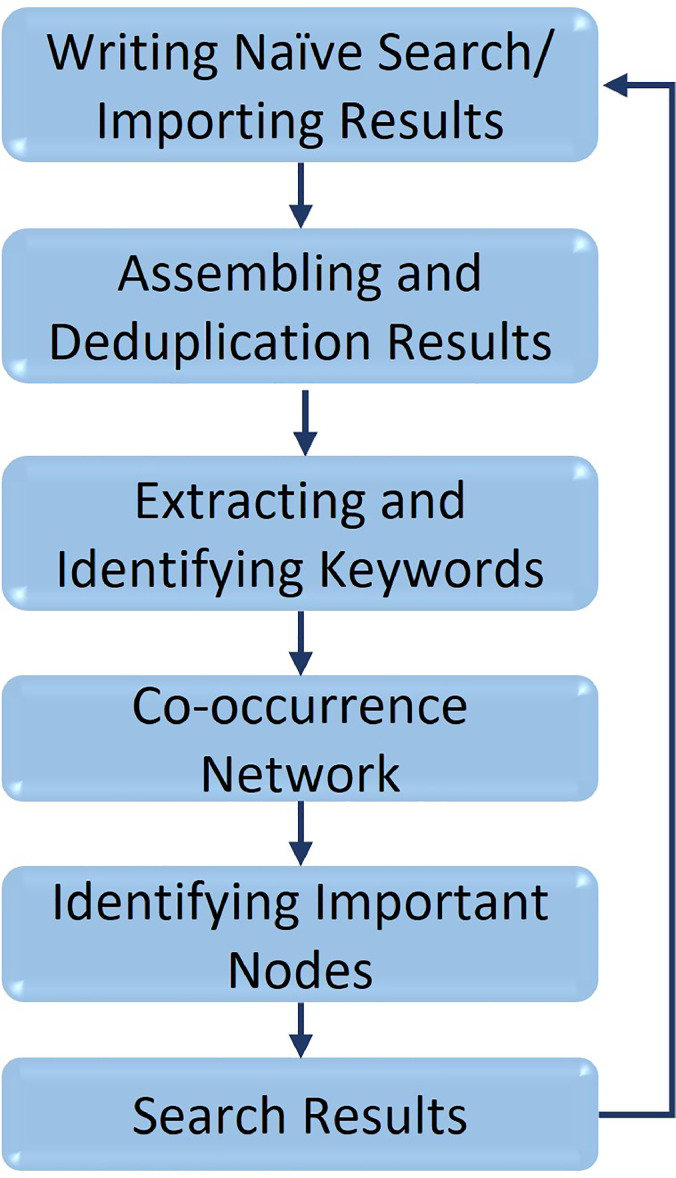
Ananse process flow

Figure 2 shows the process flow used in implementing *Ananse.*

Naïve search is written and imported. Results are assembled and deduplicated, followed by keyword extraction, creating a co-occurrence network, and identifying important nodes. After getting results, the process can be initiated for other searches.

### Software implementation and results

#### Writing the naïve search and exporting the results

When writing a naive search, the first step is to clearly articulate the research question (Grames et al., 2020). The naїve search must be precise; otherwise, it will return several unrelated articles, weakening the subsequent keyword selection [22]. The authors, who are experts in the domain of cumulative effect assessment, developed the initial search terms (76 search terms) under different concept categories to guide the identification of studies for the naïve search. We grouped the search terms into three concept categories and combined them into a Boolean search (see Table 1). Using the initial search terms of 76, we conducted a naïve literature search in three sample databases: JSTOR, Scopus, and Web of Science. These three databases were chosen to broaden the available pool of search terms on the topic as their coverage differs substantially [45].

**Table 1:** Search terms grouped under different concept categories.

***Concept category 1:***(Cumulative effect* OR Cumulative impart* OR Cumulative disturbance* OR Cumulative environmental effect*) OR (Environmental effect* OR Environmental impact*) OR (Strategic environmental assessment* OR Impact assessment*) OR (Social effect* OR Economic effects* OR Strategic effects* OR Economic Effects*) OR (Human health* OR Human Health Effects*) OR Regulatory drive OR Risk assessment* OR Systematic approach*
***Concept category 2:***forest* OR forest ecosystem* OR forest management* OR forest disturbance OR forest dynamics* OR forest growth* OR forest community* OR forest bird* OR forest land* OR forest policy* OR forest sustainability* OR forest cover OR forest carbon* OR forest soil* OR forest soil nutrients* OR forest biodiversity* OR forest conservation* OR forest structure* OR understory vegetation* OR Indigenous people livelihoods* OR Electricity generation OR forest stream* OR silviculture* OR ecosystem* OR population* OR community* OR land use/cover conversion* OR water quality* OR water quantity* OR species composition* OR endangered species*
***Concept category 3:****Mining OR Minerals and metal OR Oil and gas OR Oil sands development OR Peat mining OR Storm (wind) OR Pulp and paper industry OR Barriers OR Wildfire OR Planting OR Forest disease OR Forest health OR Forest pest OR Deforestation OR Linear features OR Electricity generation OR Roads OR Power lines OR Seismic lines OR Urbanization OR Land reclamation /restoration OR Global change OR Climate change OR Defoliation OR Insect outbreak OR water and wetlands OR Logging OR Wells OR Flood OR Drought OR Hydro development*

#### Importing naïve search results into Ananse

**Fig. 3:**
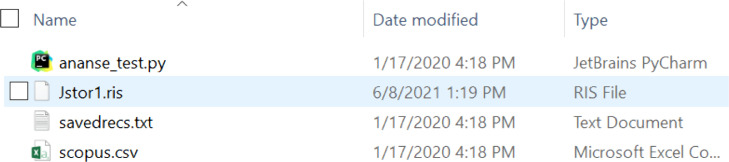
Naïve search file and results from the three databases

Ananse is a package and is provoked through a file. The naïve search results from Jstor, Scopus, and Web of Science databases were exported as an ris file, csv file, and txt file, respectively; s*; Jstor with a .ris, Web of Science* with a .csv file extension, and *Scopus* with a .txt file extension. Due to the different formats in the exportation of results from the databases, this manual process takes more time. All these three files were fed into Ananse at the same time. Using these files as input, *Ananse* merges all the different file formats into a single Pandas data frame. The merging resulted in a csv file containing 129,407 articles. Figure 3 shows the results of the naïve search and the file “ananse_test.py” that provokes Ananse to perform the search.

#### Assembling and deduplicating results

**Fig. 4:**
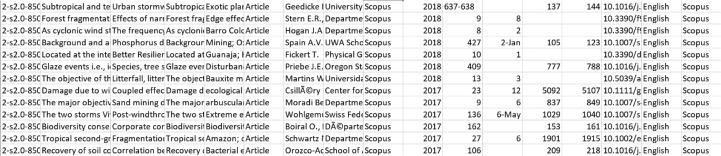
Screenshot of deduplicated files in csv format

Many articles indexed in multiple databases may pop up more than once searching for information, resulting in an overrepresentation of terms. The naïve search results were assembled and deduplicated to prevent over-representation. Provided that the path to the directory of search results is given, the *import_naive_results* function in *Ananse* automatically finds each file's database and file type, selects analogous columns, and joins them to form a single dataset. This function imports the search results from a specified path. If the parameters *clean_dataset* and *save_dataset* are set to *TRUE*, the function deduplicates search results after importing and saves the full search results to a csv file. The parameter *save_directory* contains the path to a directory where search results will be saved. If *save_dataset* is set to *TRUE* while the parameter *save_directory* is set to the directory of choice, the merged file is saved to that directory path containing the naive search results files. After the results are obtained, a *pandas* data frame consisting of assembled search results is returned. After the merging, Ananse performs deduplication based on the article titles and abstracts and returns different articles. In this instance, Ananse removes the exact title duplicates; titles that are over 95% similar or abstract that are more than 90% similar are removed. The user can change these similarity levels. Ananse returned 6,786 distinct articles out of the 7,809 articles fed into it and created a csv file, a screenshot of it is as shown in Fig. 4 (the content of the csv file is available in the appendix). Ananse automatically corrected and classified 100% of the 1023 duplicate articles identified by manual deduplication.

#### Extracting and identifying keywords

Ananse uses the Rapid Automatic Keyword Extraction (RAKE) [35], a keyword extraction method, to extract potential keywords from the titles, keywords and abstracts of articles in the deduplicated dataset. The *RAKE* is designed to identify keywords in scientific literature by selecting strings of words uninterrupted using a list of stopwords (6+) and phrase delimiters (punctuation) to detect the most relevant words or phrases in a piece of text [36]. The function *extract_terms* call the RAKE algorithm and eliminates keywords that only appear in a single article and excludes phrases with only one word from the list of potential keywords resulting in a more precise search. *Ananse* then combines the author- and database-tagged keywords with the search terms. The author and database tagged keywords are combined as dictionary objects created with *extract_terms* to define all possible keywords. All the possible keywords are then passed to a function *create_dtm* for function wrapping, which generates a *document-feature matrix* using the potential keywords as features and the combined titles, abstracts, and keywords of each article (also referred to as noted) as the documents.

#### Co-occurrence network

The selection of keywords using the frequency of occurrence can be a good indicator of the relevance of a word/term to a search strategy. However, we moved beyond this and generated a keyword co‐occurrence network. The co-occurrence network creates and measures each term's importance and influence in relation to the topic being reviewed [37]. Using the document matrix containing the potential keywords, we generated a keyword co-occurrence network. Each keyword is represented by a point referred to as the node, and an edge also represents a link between the keywords. Each node represents a potential search term, and the edges are co-occurrences of two terms in a study's title, abstract, or tagged keywords [37]. In *Ananse*, the co-occurrence network is implemented with the function *create_network,* which measures the importance of each term in relation to the selected topic being reviewed. The function *get_centrality* is used to evaluate the node importance of a graph and returns a dictionary containing nodes with their importance.

**Fig. 5:**
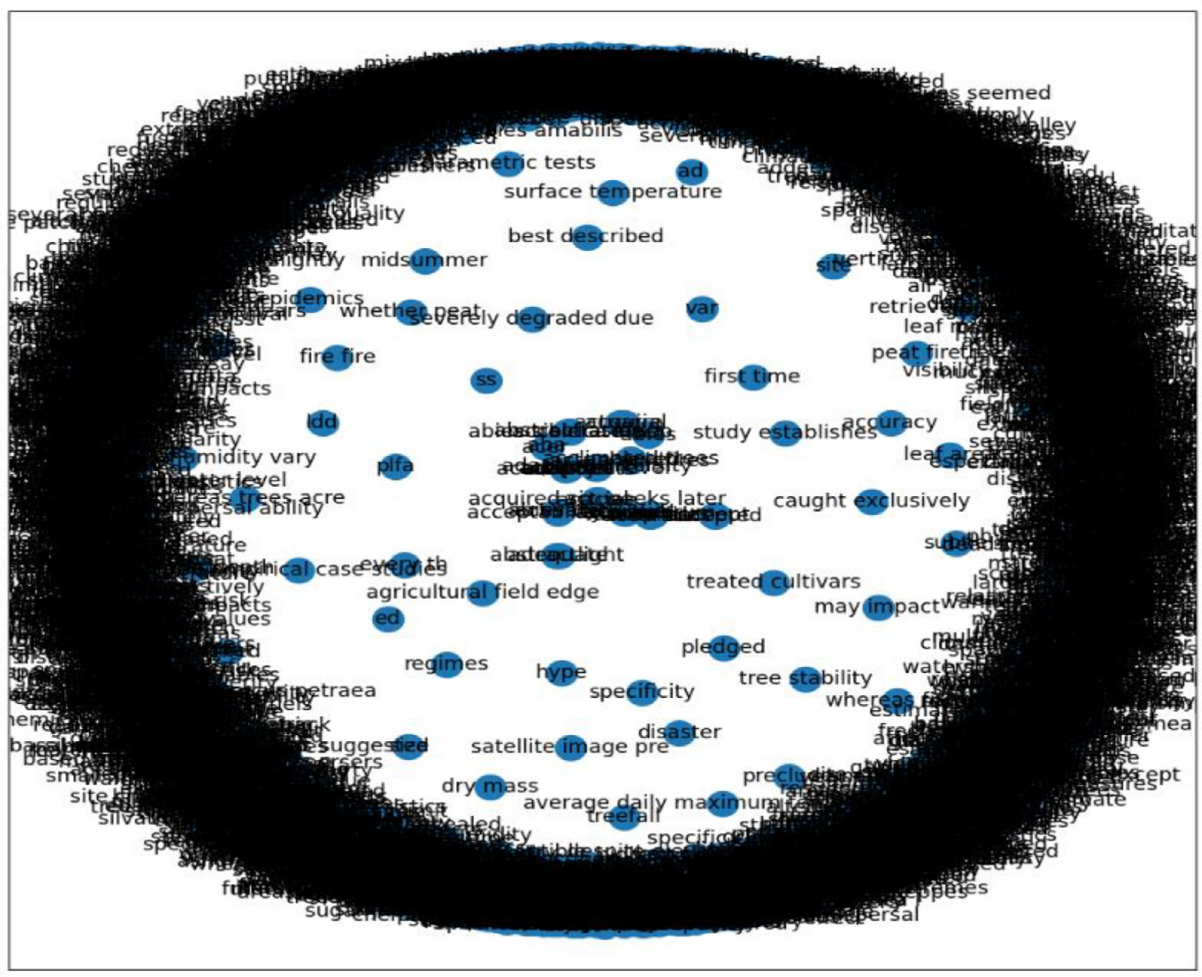
Co-occurrence Network from the case study

Figure 5 shows a co-occurrence network with important keywords closely grouped. The dense region shows keywords that are closely related.

#### Identifying important nodes using a full network

**Fig. 6:**
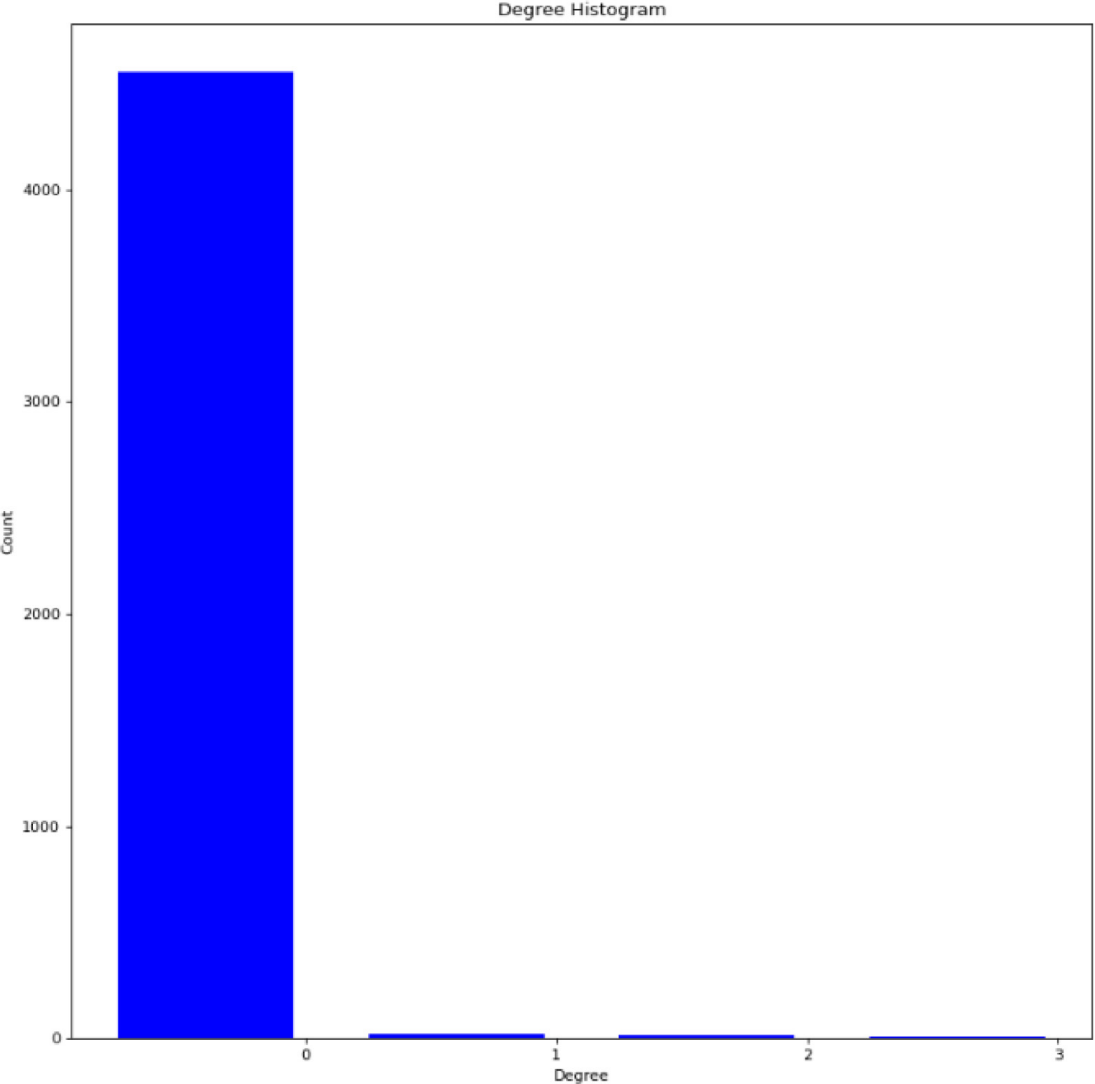
Degree Histogram of degree and counts

Important nodes represent keywords to be used to generate final search terms. Two methods to identify important nodes were explored in *Ananse*: fitting a *spline model* to the node importance to select tipping points and *cumulative approach,* which finds the minimum number of nodes to capture a large percentage of the total importance of the network. One can decide which method to use depending on the distribution and preference. In choosing a method, the first thing to do is to look at the distribution of node importance. In Ananse, the distribution was plotted with the function *plot_degree_distribution, plot_rank_degree_distribution,* or *plot_degree_histogram* as shown in Fig. 6

**Fig. 7:**
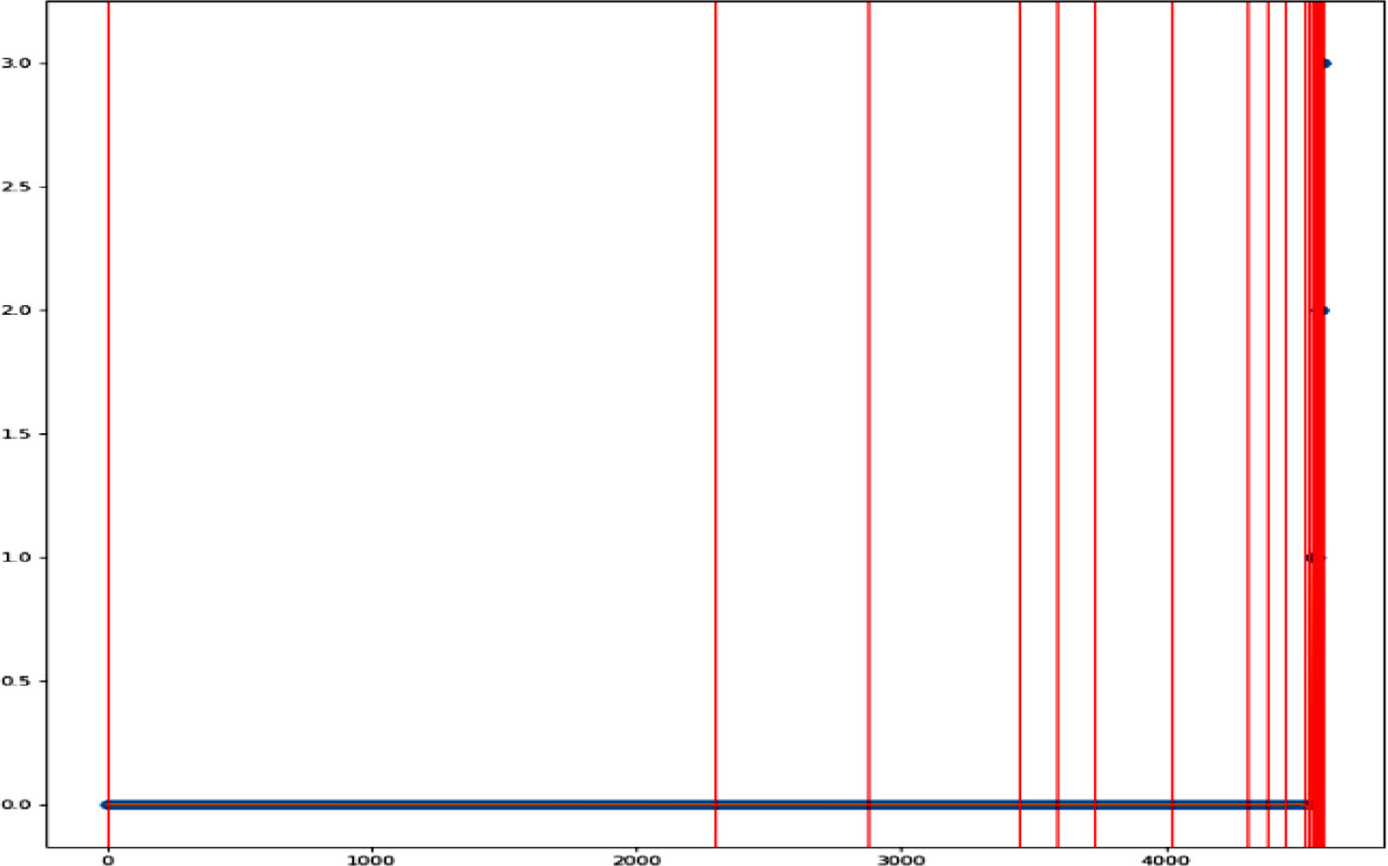
(Ranked Node Strength with cut-off points)

A spline model for finding cut-off is an appropriate method to identify the cut-off threshold for keyword importance if the rank distribution plot has a lot of weak nodes with a long tail. On the other hand, the cumulative approach is more appropriate when there are no clear breaks in the data. In Ananse, the *find_cutoff* function finds the *cut-off* for a graph network using either cumulative or spline method of cutting the degree distribution, as shown in Fig. 7. The *reduce_graph* function then generates a graph consisting of only important nodes, after which the *get_keyword* function extracts the keywords from the reduced network.

**Fig. 8:**
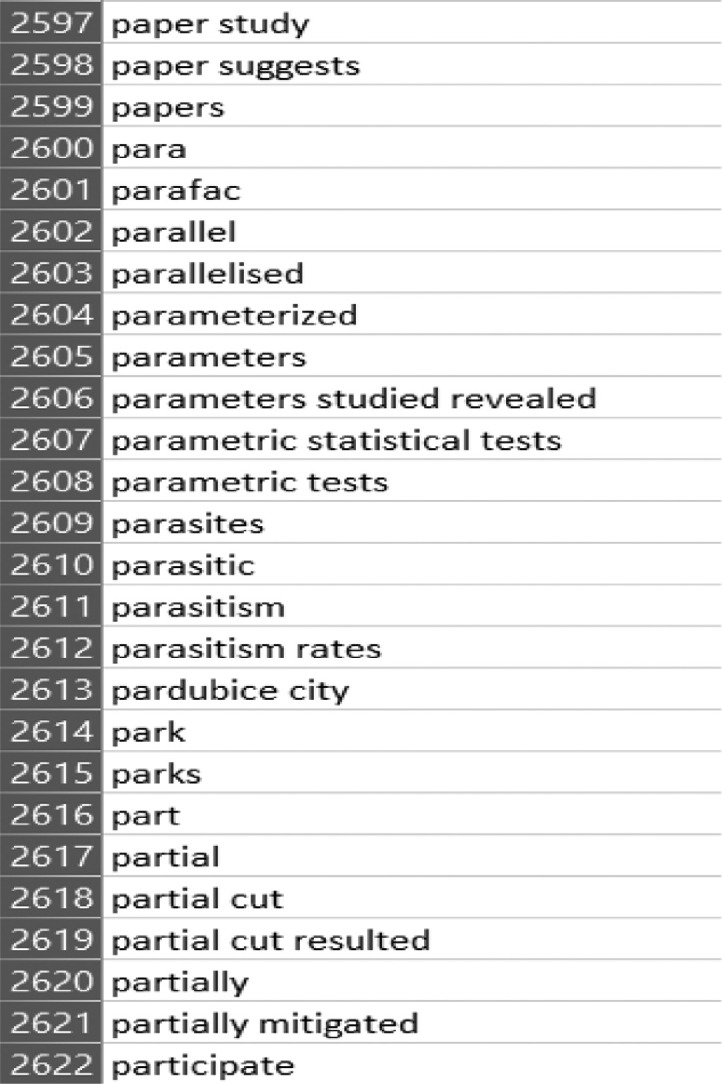
A section of relevant keywords.

Ananse uses the node strength to generate relevant keywords from which the experts can now select their final keywords. In this research, Ananse generated 4,596 keywords. A screenshot of it is shown in Fig. 8 (the content of the csv file is available in the appendix). Afterward, the researchers manually reviewed each word or phrase using their expert knowledge to arrive at the final keywords.

The final list of search terms (listed as search strings) was grouped under three concepts, as shown in Table 2. These concepts (and terminology) are cumulative effects, forests and forest ecosystems, and types and forms related to forest disturbance [30].

**Table 2:** Final list of search terms.

Concept A: Cumulative effets terminologies	Concept B: Resource development/disturbance	Concept C: Forest landscape dynamics
Cumulative effect	Mining	forest
Cumulative impact	Minerals and metal	forest ecosystem
Environmental effect	Oil and gas	forest management
Environmental impact	Oil sands development	forest disturbance
Cumulative disturbance	Peat mining	forest dynamics
Impact assessment	Storm (wind)	forest growth
Cumulative environmental effect	Pulp and paper industry	understory vegetation
Social effects	Barriers	forest community
Economic effects	Wildfire	forest bird
Strategic environmental assessment	Planting	forest land
Risk assessment	Forest disease	Indigenous people livelihoods
Systematic approach	Forest health	forest policy
Human health	Forest pest	forest sustainability
Human Health Effects	Deforestation	forest cover
Regulatory drive	Linear features	forest carbon
	Electricity generation	landscape
	Roads	forest stream
	Power lines	silviculture
	Seismic lines	ecosystem
	Urbanization	population
	Land reclamation /restoration	community
	Global change	land cover conversion
	Climate change	water quality
	Defoliation	water quantity
	Insect outbreak	forest soil
	water and wetlands	forest soil nutrients
	Logging	forest biodiversity
	Wells	forest conservation
	Flood	forest structure
	Drought	species composition
	Hydro development	endangered species
	Habitat fragmentation	forest habitat
	Landscape fragmentation	wildlife
	Species invasion	soil compaction
	Urban expansion	soil porosity
	Habitat alteration	soil quality
	Loss of biological diversity	functional traits
	Soil acidification	Forest soil biodiversity
	Forest harvesting	
	Air Pollution	
	Water pollution	

## Discussion

Evidence synthesis has become an essential feature of the current academic landscape, although a lack of transparency often hampers the process. This research reports on the methods used to select search terms that form the building block for performing evidence synthesis and offers a transparent approach to understand underlying assumptions. In systematic reviews, the selection of key search terms is considered the basic building block for the successful assemblage of knowledge in a particular field. However, this process is often left to researchers' discretion, leaving room for biases and a subjective selection process, affecting the outcomes of effective evidence synthesis. In this research, we designed and implemented a partially automated keyword search software package using Python for SR to enhance efficiency, maximize transparency and comprehensiveness while minimizing subjectivity and bias. Dubbed *Ananse*, our tool provides an efficient and standardized method for developing search strategies using NLP and co-occurrence networks to identify relevant search terms.

Our approach combines expert knowledge with a quasi‐automated method which enhances search recall. This is very important for fields such as ecology, where non‐standardized or nuanced terminology or a lack of formal ontologies exist for conducting SRs [22]. Most importantly, Ananse significantly reduces the time required to conduct a SR by decreasing time spent on search strategy development and tedious tasks like assembling and deduplication. Compared with the manual process of assembling results, Ananse reduced by more than half the time required to assembly results. Similarly, while it took two of the co-authors two days of full-time work to remove duplicates, Ananse removed the duplicates efficiently in about a minute or less and achieved 100% accuracy. With the reduction in time needed to develop a search strategy and assemble and deduplicate the results, our approach makes extensive systematic reviews and meta‐analyses more efficient and effective compared with conventional approaches. Our research contributes to the emergence and application of an ever-growing set of tools and software that can be used to facilitate transparent, reproducible reviews and develop reproducible synthesis workflows such as metaDigitise [38], litsearchr [22] in R, and revtools [39]. These efforts should help facilitate the reproducibility of ecological reviews, enhance transparency, and improve the rigor of evidence used to guide policy decisions [40].

In its current implementation*, Ananse,* a Python package, contains a suite of functions to improve the efficiency of keywords selection for systematic reviews. For instance, by automatically deduplicating and assembling results from separate databases, Ananse provides a systematic approach to facilitate knowledge synthesis through SR. Also, apart from generating keywords, it can act as middleware or a data converter for integrating multiple datasets into a database. Done manually, this is a time‐intensive process because platforms and databases export results in different formats [2]. Furthermore, we used the agile method of software engineering with open-source software development, thereby making *Ananse* easily customizable and improved upon as researchers use it beyond the application to cumulative effects assessments. Currently, Ananse has a popularity of 131 downloads per week on the Python Package Index (https://snyk.io/advisor/python/ananse). Ananse contributes to the development of open-source software systems needed to speed up systematic review. In its current state, Ananse provides a means to merge and deduplicate keywords for experts programmatically. By its design and implementation, Ananse allows researchers to modify their requirements without creating new software. Even though Ananse has been used for a cumulative effect use case [30], it is general-purpose software for a systematic review of any kind. It can be applied broadly in ecology and evolutionary biology as well as other fields.

## Conclusion

Compared to conventional approaches for developing keywords for systematic review, our method is far effective and efficient by significantly reducing the time and resources needed to develop search strategies to conduct systematic reviews. *Ananse* substantially reduces the time spent on the systematic review by automating time-consuming tasks such as assembling and deduplicating large search results. Ananse saves time and enhances effective keyword generation compared to traditional methods by automating the tedious and bias-prone aspect of systematic review tasks. Therefore, *Ananse* presents an approach to performing large systematic reviews within a short period of time.

Our results can be used as a starting point to frame future studies according to well-defined terminology. Future research would enhance the front-end of Ananse through a user-friendly graphical interface. Currently, Ananse allows one user per time; this functionality can be improved by making Ananse a server-type software with capabilities to permit concurrent and multi-user interaction. The requirements would be modified as we get feedback from the research community.

## Software, data, and documentation availability

The source of this software is publicly available via Github [41] and also via PyPI [42]. Documentation is accessible via [43] and [44].
